# Interleukin-10 augments human endogenous retroviral E1B variant of *cd5* in aged T cells

**DOI:** 10.1007/s44313-025-00080-8

**Published:** 2025-08-11

**Authors:** Bharat Singh, Smita Kumari, Amit Kumar Kureel, Arunim Shah, Shobhita Katiyar, Chandra Prakash Chaturvedi, Kulwant Singh, Ambak Kumar Rai

**Affiliations:** 1 Department of Biotechnology, Motilal Nehru National Institute of Technology Allahabad, Prayagraj-211004 (U.P.), India; 2 Stem Cell Research Center, Department of Hematology, Sanjay Gandhi Postgraduate Institute of Medical Sciences, Lucknow-226014 (U.P.), India

**Keywords:** Aging, Surface CD5 (sCD5), HERV, IL-10, CEBP-β/LIP

## Abstract

**Purpose:**

Aging leads to immune dysfunction, including altered T-cell phenotypes such as the CD5^low^ state. This study investigated how the exon switch regulates CD5 expression in aging in an interleukin-10 (IL-10)-dominated environment and the involvement of CCAAT/enhancer-binding protein beta (CEBP-β) in this process.

**Methods:**

The expression of messenger RNA (mRNA) was analyzed for E1A and E1B in T cells from young and older adults. The effect of IL-10 treatment on the exon switch was assessed by measuring the E1A and E1B mRNA expression in young T cells. MatInspector analysis identified CEBP-β binding sites upstream of E1A and E1B start sites. The effect of IL-10 on CEBP-β isoforms expression was assessed using western blot, and that on CEBP-β binding onto the E1A and E1B upstream was assessed using chromatin immunoprecipitation assays. The short hairpin RNA (shRNA) silencing of CEBP-β was performed to confirm its role in E1A/E1B expression.

**Results:**

Older individuals showed increased E1B and decreased E1A mRNA expression. IL-10 treatment of young T cells persuaded a similar shift. IL-10 changed CEBP-β binding, reducing its association with the E1B upstream region while increasing its binding to E1A. IL-10 also upregulated the liver-enriched inhibitory protein of CEBP-β. shRNA silencing of CEBP-β reduced E1B expression.

**Conclusion:**

IL-10-driven exon switching alters CD5 expression in aged T cells, increasing E1B and decreasing E1A through CEBP-β regulation. These findings reveal a novel mechanism underlying fundamental immune aging and suggest potential targets for immune modulation. These insights may have clinical implications in chronic inflammatory diseases, autoimmune disorders, and cancer therapies.

**Supplementary Information:**

The online version contains supplementary material available at 10.1007/s44313-025-00080-8.

## Introduction

The immune response in older adults is not as robust as that in younger individuals, and the risks of immune disorders, autoimmune diseases, and poor immune response against infectious diseases are increased. Aging of the immune system (i.e., immune aging) is characterized by multiple age-related changes resulting in varying immune responses to any new or recurring antigens. Some of these changes include diminished production of immune cells (B and T cells) in primary lymphoid tissues and reduced functioning of mature lymphocytes in secondary lymphoid tissues [1, 2]. In addition, these lymphocytes show a less efficient response to recall antigens in older individuals than in younger individuals; however, lymphocytes are continuously produced in older individuals [3]. In older individuals, the number of lymphoid hematopoietic stem cells (HSCs) decreases, and that of myeloid HSCs increases, resulting in a low number of lymphoid progenitor cells [4].

Older individuals show a higher level of apoptosis and slower proliferation of T-cell progenitors within the thymus than younger individuals [1, 5]. An increased number of CD8^+^ memory T cells with reduced CD28 levels and impaired cytolytic function is considered a hallmark of immune aging [6, 7]. Furthermore, aged CD4^+^ T cells exhibit a less competent phenotype (CD28^low/neg^) with diminished T-cell receptor (TCR) signaling and abrupt cytokine production following antigen presentation [8, 9]. B-cell insufficiency has also been correlated with T cell deficiency, because CD4^+^ T cells play a critical role in coordinating the functions of other immune cells [10, 11]. When competent T cells interact with antigen-presenting cells, this interaction dictates the fate of T cells, which is regulated by their surface receptors, such as CD6 and CD5. Therefore, exploring their role in T cells may provide valuable evidence to boost immune competence in older individuals [12].

CD5 is a surface glycoprotein that plays multiple roles in T-cell function, including development, activation, proliferation, and TCR signaling [13–16]. It is expressed by thymocytes, mature T cells, and a small B-cell population (B1a cells). CD5 expression is regulated during T-cell development and activation [17]. Any reduction in expression can significantly affect T-cell function [18]. Diminished expression of surface CD5 (sCD5) is associated with several pathological conditions, including systemic lupus erythematosus [19], multiple sclerosis [20], B-cell chronic lymphocytic leukemia [21], and T-cell acute lymphoblastic leukemia (T-ALL) [22]. The *cd5* gene has two transcript variants: E1 A (NM_014207.4) and E1B (NM_001346456.2). The E1B variant has an alternative transcription start site (TSS) derived from a 5254 bp long integrated sequence of human endogenous retroviral origin [23]. The non-conventional promoter of the E1B variant is located 8.2 kb upstream of the conventional TSS (E1 A TSS) of the *cd5* gene [24]. This promoter contains various transcription factor (TF)-binding sites (TFBSs) that regulate the expression of E1B and eventually affect sCD5 expression. Additionally, the switch from exon E1 A to E1B was directly associated with reduced sCD5 and cytoplasmic CD5 (cCD5) protein accumulation in T and B cells. In 2017, our group showed a transcriptional/exon switch from a conventional E1 A start site to a non-conventional E1B start site, leading to a shift from E1 A mRNA (sCD5↓) to E1B messenger RNA (mRNA) (cCD5↑) in human T cells. This explains the CD5^neg/low^ phenotype of leukemic T cells.

Immune aging is characterized by abrupt cytokine production followed by inflammation, which is defined as inflammaging. Inflammaging is characterized by increased interleukin-10 (IL-10) [25–27]. Persistent inflammation reduces the cellular capacity to respond to new and recall antigens. In another study, Tatari et al. [28] showed reduced sCD5 expression in aged T cells in mouse models. Therefore, we investigated the relationship between increased IL-10 levels and decreased CD5 expression during aging. In the present study, we identified regulatory factors of CD5 expression by in silico analysis of the TFBS of the E1 A and E1B promoters of the *cd5* gene. We found a CCAAT/enhancer-binding protein beta (CEBP/β) binding site in the promoter region, which is linked to aging-related processes, and its enrichment on these sites is also increased by IL-10 cytokine. Aging-related chronic inflammation, or “inflammaging,” has been widely associated with T-cell dysfunction. Our findings suggest that the E1 A → E1B exon switch in CD5 represents a key molecular event contributing to T-cell senescence and T-cell function. This shift, influenced by IL-10 and the liver-enriched inhibitory protein (LIP) isoform of CEBP-β, reduces sCD5 expression, potentially altering immune responsiveness and increasing susceptibility to age-related immune dysregulation.

## Materials and methods

### Recruitment of healthy young and older volunteers

Blood samples from healthy young (*n* = 20; age: mean ± standard deviation [SD], 27.67 ± 2.7278; M/F, 10/10] and older (*n* = 20; age: mean ± SD, 67.79 ± 7.046; M/F, 11/09) individuals with no history of chronic illness and antibiotic/steroidal treatment for the last three months were collected. Informed consent forms were obtained from all participants, and they were informed of their involvement in the process. Blood samples were collected in EDTA vacutainers by trained paramedical staff at the Institute Health Centre, Motilal Nehru National Institute of Technology, Allahabad. This study was approved by the institute’s ethics committee (MNNIT-IEC: Ref. No. IEC/2019–20/06) and strictly adhered to the guidelines. Demographic details of the participants are presented in Table 1.

**Table 1:** Demographic characteristics of human participants included in the study

Older individuals (*n* = 20)	
**Demographic characteristic**	
Age (mean ± SD, range)	67.79 ± 7.046 (60–86)
Sex (M/F)	11/9
Ethnicity	Indian
Region	Uttar Pradesh, India
**Other diagnoses**	
Total leukocyte count (cells/mm^3^) (mean ± SD, range)	8528.5 ± 1999.9634 (3600–12800)
Neutrophil (%) (mean ± SD, range)	62.596 ± 7.0223 (51.00–78.00%)
Lymphocyte (%) (mean ± SD, range)	29.92 ± 6.0883 (17–40%)
Eosinophil (%) (mean ± SD, range)	4.145 ± 2.1232 (2–9%)
Monocyte (%) (mean ± SD, range)	2.43 ± 1.7867 (1–8%)
Basophils (%) (mean ± SD, range)	0.00 ± 0.00 (0%)
Random blood sugar (mg/dL) (mean ± SD, range)	104.524 ± 15.63 (75–144.2)
Serum creatinine (mg/dL) (mean ± SD, range)	0.967 ± 0.1753 (0.70–1.21)
Hemoglobin (g/dL) (mean ± SD, range)	11.67 ± 1.1995 (7.8–13.4)
**Young individuals** (*n* = 20)	
**Demographic characteristic**	
Age (mean ± SD, range)	27.67 ± 2.7278 (24–36)
Sex (M/F)	10/10
Ethnicity	Indian
Region	Uttar Pradesh, India
**History (young and older individuals)**	
**Any steroid taken**	No
**Other diseases/chronic illnesses**	Not diagnosed/not reported

### Isolation of human peripheral blood mononuclear cells (PBMCs)

PBMCs were isolated from whole blood using the Ficoll-Hypaque density gradient centrifugation method [29]. Blood samples were mixed with 1 × phosphate-buffered saline (PBS) in a 1:1 ratio. The mixture was layered on top of the Ficoll-Hypaque solution (HiSep™ LSM 1077; HiMedia; ratio: 2:1, diluted blood: Ficoll solution) in a 15 mL centrifuge tube. The layered mixture was centrifuged at 2000 rpm for 20 min at room temperature. PBMCs were present in the buffy coat interphase layer below the plasma layer. The buffy coat was retrieved and washed three times with 1 × PBS. The washed PBMCs were then suspended in complete RPMI (31,800–022, Gibco Life Technologies, with L-glutamine, without HEPES and NaHCO3) with 10% fetal bovine serum (FBS) (10,270,106, Gibco Life Technologies). Cell viability was assessed using trypan blue dye (0.3%; Cat. No. T8154, Sigma) exclusion assay, and samples with more than 95% viability were considered for further experiments.

### PBMC culture and IL-10 treatments

The PBMCs were cultured in complete RPMI (i.e., with 10% FBS) at a cell concentration of 2 × 10^6^ cells/mL in a 5% CO_2_ incubator for 24 h at 37 °C. The PBMCs were treated with 30 ng/mL recombinant IL-10 (Cat. No. BAN2142, ImmunoTag, USA) for 24 h, according to the experimental plan. At the end of the incubation period, the cultured cells were washed once before proceeding to the next step of the experiment.

### Isolation of total RNA and complementary DNA (cDNA) synthesis

Total RNA was isolated using the TRIzol assay, as described by Kureel et al. [30]. The quality and integrity of the isolated RNA were assessed using 1% agarose gel electrophoresis in nuclease-free diethylpyrocarbonate-treated water. The RNA purity and concentration were evaluated using a microvolume spectrophotometer (Picogene™, GX-SX2-AG, Thermo Scientific, USA). DNA contamination was removed using DNase I (EN0521, Thermo Scientific, USA) digestion assay.

cDNA from RNA was synthesized using M-MLV reverse transcriptase (M0368L, New England BioLab, USA), OligodT (S0131, Thermo Fisher Scientific, USA), random hexamers (S0142, Thermo Fisher Scientific, USA), Ribolock™ RNase inhibitors (EO0381, Thermo Fisher Scientific, USA), and dNTP (R0192, Thermo Fisher Scientific, USA). For cDNA conversion of 500 ng of total RNA, all reagents were used according to the manufacturer’s protocol. Thermal cycler conditions were maintained as required for the optimal activity of M-MLV reverse transcriptase.

### Quantitative polymerase chain reaction (qPCR) analysis for the gene of interest

Real-time qPCR was performed to analyze the mRNA expression levels of the genes of interest. Five microliters of high-fidelity SYBR green reaction mixture (F415L, Thermo Fisher, USA) was mixed with gene-specific reverse and forward primers (0.5 µM each) along with 1 µl of respective cDNA samples, and the final volume was adjusted to 10 µl. The mixture was subjected to thermal cycling conditions in Applied Biosystems™ StepOne™ Real-Time PCR System (4,376,357, Thermo Fisher Scientific, USA). The annealing temperature for each primer set was optimized before performing qPCR. U6 was used as the housekeeping control for mRNA expression analysis. The obtained cycle threshold (Ct) values were normalized using housekeeping control, and relative fold change analysis was done using the double delta Ct (2^−∆∆Ct^) method [31]. The primer sequences are listed in Table 2.

**Table 2:** List of specific primer sequences used in the study

Name of the primer	Accession no	Primer sequence	Tm
CD5 E1 A mRNA primer	NM_014207.4	FP: GAGGCAAGAGAAGGCCAGAAAC RP: TTGCCTGGAAATCTGGGTCATA	53 °C
CD5 E1B mRNA primer	NM_001346456.1	FP: TGACCAGGAAGCAAAGTGATTA RP: AGGAAGCGACAAGTTTCAGT	59 °C
CEBP-β mRNA primer	NM_005194.4	FP: GACGAGTACAAGATGCGGC RP: TGCTTGAACAAGTTCCGCAG	54 °C
U6 mRNA primer	NR_004394.1	FP: GTGCTCGCTTCGGCAGCACATATAC RP: AAAAATATGGAACGCTTCACGAATTTG	61 °C
CD5 E1B ChIP DNA primer E1B_NONLTR_1	–	FP: CCAGCCTGAGATGAGAGAGA RP: AACAGTGAGCTTTGGACTGG	58.5 °C
CD5 E1B ChIP DNA primer E1B_NONLTR_2	–	FP: GCACCTTGGAGAATTCACATAAC RP: CAGGTGTTAATGAAGGCTGTTG	58.5 °C
CD5 E1B ChIP DNA primer E1B_NONLTR_3	–	FP: GGGCCGAATACTTTACCTTCT RP: GCTCCTTCAGTCTTCCCAATTA	58 °C
CD5 E1B ChIP DNA primer E1B_LTR_1	–	FP: ACCTAATCGGTTATGTCATCTAT RP: GCATGCACCGGTAATTAGAA	55 °C
CD5 E1 A ChIP DNA primer E1 A_1	–	FP: CAGCCATCTCTTGTACTTGCT RP: AGGGATGAGGTTGGAAGTTTG	58 °C
CD5 E1 A ChIP DNA primer E1 A_2	–	FP: AGATCACACACAACACAGAAA RP: TGTGTGAACTCCATGTTGATG	55 °C
CD5 E1 A ChIP DNA primer E1 A_3	–	FP: GAGATCACATTCACACACACAAAC RP: TGTATGATCTCCATGTTGACT	57.5 °C

### Prediction of TFBSs

The DNA sequence of 5 kb upstream of the TSS of the non-conventional *cd5* E1B transcript variant and 1.1 kb upstream of the *cd5* E1 A TSS was retrieved using the UCSC genome browser. The nucleotide sequence was uploaded to MatInspector software version 8.1 of the Genomatix suite v3.4 [32]. A TFBS analysis was performed. TFBS analysis provided a score-weighted list of DNA motifs in the promoter sequence. The results were downloaded from a PDF containing detailed information on all the identified TFBSs, such as the matrix family, anchor position, target sequence, and similarity score.

### Short hairpin RNA (shRNA) mediated CEBP-β silencing transfection

Two shRNA targeting coding sequence (CAAGGCCAAGATGCGCAACCT) and 3′UTR (CCCGTGGTGTTATTTAAAGAA) region of CEBPb mRNA were cloned in a lentiviral vector plko.1-puro (Addgene plasmid #8453). Negative control shRNA (containing a scrambled sequence as the target sequence; TRC) was used as a control. For lentiviral packaging, pLKO-shRNA was co-transfected with pCMV-dR8.91 and pCMV-VSV-G into 293 FT cells using Lipofectamine 3000. The culture supernatant containing the lentiviruses was collected 48 h after transfection. The viral supernatants were harvested, centrifuged at 1000 g for 10 min to remove the cell debris, and stored at 4 °C. Freshly isolated PBMCs from young and healthy individuals were then transduced with lentiviral particles packaging scrambled or specific shRNA targeting CEBPb. Briefly, 2 × 10^6^ PBMCs were co-cultured with lentiviruses in a complete medium containing polybrene (12 µg/mL) at 37 °C in a CO_2_ incubator. The transduced cells were selected in complete media containing 7 µg/mL of puromycin for 24 h before the cells were used for further experiments. This study was conducted at SGPGIMS, Lucknow, U.P., India.

### Preparation of protein lysate

Protein lysates from cultured cells were prepared using Laemmli buffer [for 2 × : 2% sodium dodecyl sulfate (151–21-3, Thermo Fisher Scientific, USA), 30% (w/v) glycerol (62,417, SRL Pvt. Ltd.), and 72.5 mM Tris–HCl (99,438, SRL Pvt. Ltd.); pH 6.8] [33]. Cultured cells were washed twice with 1 × PBS, and 2 × concentrated Laemmli buffer was added. Laemmli buffer helps release the complete intracellular content outside the cells. The busted cell suspension in Laemmli was kept on ice for 10 min, followed by heat treatment at 95 °C for 10 min. The prepared lysate was stored in aliquots at −80 °C for further analysis.

### Western blotting analysis

Cell lysates prepared in 2 × Laemmli sample buffer were subjected to denaturing sodium dodecyl sulfate–polyacrylamide gel electrophoresis. Next, 4% stacking and 12% resolving polyacrylamide gels were used to separate the proteins of interest. After the run, the proteins were transferred from the gel to a 0.22 µm polyvinylidene fluoride (PVDF) membrane (SVFX, 0.2 µm, MDI Membrane Technologies) in a semi-dry blotting membrane transfer system (120059 GB, Merck Life Sciences). After transfer, the membrane with the protein imprint of the colored marker from the gel was blocked using a solution of 5% bovine serum albumin (BSA) and 5% non-fat dry milk. Proteins of interest were detected with anti-human CEBP-β polyclonal antibody (ITT0553, C/EBP/β polyclonal antibody, ImmunoTag, USA), anti-human GAPDH monoclonal antibody (HRP-60004, HRP GAPDH monoclonal antibody, Proteintech, USA), and anti-mouse IgG-FITC antibody (sc-2010, Santa Cruz Biotechnology). An iBRIGHT CL-1000 imaging system (Thermo Fisher Scientific, USA) was used to visualize the blots on a PVDF membrane.

#### Chromatin immunoprecipitation (ChIP) assay of predicted TFBSs

The IL-10-treated and untreated cells were harvested, washed with 1 × chilled PBS, and fixed with 0.5% formaldehyde (TC557M, 37% formaldehyde, HiMedia) for 10 min at room temperature. A 1 M glycine solution was added to quench the action of formaldehyde. Cells were centrifuged at 2000 × g for 5 min at 4 °C, and the pellet was washed twice with 1 × PBS buffer. The pellet was lysed in 1 mL RIPA lysis buffer (R0278, Sigma-Aldrich, USA) for 10 min on ice and centrifuged at 12,000 rpm for 5 min at 4 °C. The pellet was again solubilized in 300 µl RIPA buffer. RIPA buffer was added with 1 × protease inhibitor cocktail (G6521, Promega Biotech) and 10 mM phenylmethylsulfonyl fluoride before use. Chromatin shredding was performed using a probe sonicator (JY92-IIN, Ultrasonic Homogenizer, Helix Biosciences) for 30 cycles (30 s on and 30 s off) at 40 Hz and 40% saturation. Chromatin fragments (sheared chromatin) were collected in the supernatant by centrifuging at 12,000 g for 10 min at 4 °C.

In parallel, antibody-conjugated magnetic beads targeting CEBP-β and IgG isotype antibodies were freshly prepared according to the manufacturer’s protocol. An isotype control was used to eliminate false-positive results. Chromatin sheared in RIPA buffer was incubated with the prepared antibody-conjugated magnetic beads. The mixture was rotated for 2 h at 4 °C using a rotor spin (3071, Rotospin, Tarsons, USA). For blocking, 1% BSA (GRM3151, HiMedia) was used. Magnetically separated immunoprecipitates were treated with Proteinase K (1,073,393, Sigma-Aldrich, USA) and RNase A (EN0531, Thermo Fisher Scientific, USA) to degrade all proteins and RNA contamination, respectively. DNA fragments were collected and purified using the QIAquick PCR Purification Kit (28,104). Real-time PCR was performed for all the identified TFBS upstream of exons E1 A and E1B using specific primer sets. Ct values were obtained, and the fold change was calculated using the respective isotype controls.

## Results

### Increased expression of E1B mRNA, i.e., a non-conventional variant of the *cd5* gene in older individuals

**Fig. 1:**
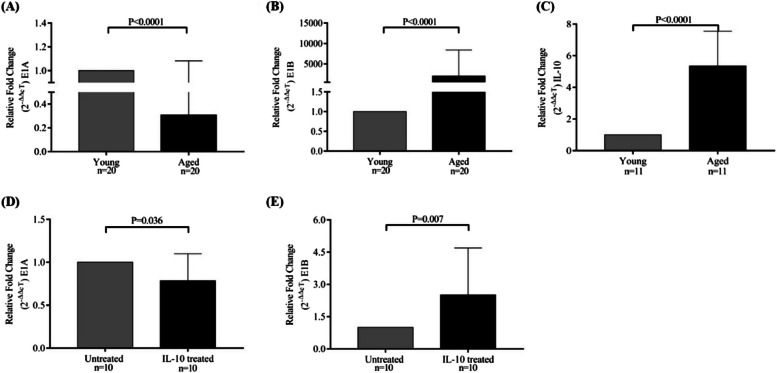
Increased ***cd5*** E1B variant in older individuals: The bar diagram shows relative fold change in (**A**) E1 A and (**B**) E1B expression of the *cd5* gene in peripheral blood mononuclear cells (PBMCs) of healthy young and older individuals (*n* = 20). C The bar diagram shows relative fold change in IL-10 mRNA expression in PBMCs of healthy young (*n* = 11) and older (*n* = 11) individuals. **D**-**E** IL-10-treated PBMCs from healthy young (*n* = 10) and older (*n* = 10) individuals show relative fold change in expressions of E1 A (D; unpaired t-test, *p* = 0.036) and E1B (E; unpaired t-test, *p* = 0.007) variants of the *cd5* gene. Expression of U6 is used as a reference gene for calculating relative fold change in expression (2^−ΔΔcT^). Significance is shown as a *p*-value using the paired t-test, and values less than 0.05 are considered significant (representative of 95% CI). Control groups (young and untreated) were normalized to 1

We initially investigated the expression of E1 A and E1B mRNA variants of the *cd5* gene in healthy young (*n* = 20) and older individuals (*n* = 20). Their relative expressions showed significantly decreased E1 A and increased E1B expressions in PBMCs of older individuals than those of healthy young individuals (for relative expressions, 2^−ΔΔcT^; E1 A and E1B, Fig. 1A and 1B, respectively). The relative fold change in expression showed a manifold increase in E1B expression in older individuals.

### Increased IL-10 in older individuals may upregulate the E1B variant of the *cd5* gene

As discussed in the Introduction, IL-10 has been documented in immune aging, particularly in age-associated immune modulation and disorders. Older individuals show elevated anti-inflammatory cytokine levels [34, 35]. Therefore, we investigated the mRNA expression of IL-10 in healthy young and older individuals. The findings showed increased IL-10 expression in the PBMCs of older individuals recruited in our study compared with that of young individuals (Fig. 1C; unpaired t-test, *p* = 0.0001). Intrigued by this finding, we cultured PBMCs from healthy young individuals with recombinant IL-10 (30 ng/mL) and measured the expression of E1 A and E1B. The finding showed a significant decrease in E1 A mRNA expression and a significant increase in E1B mRNA expression, suggesting reduced sCD5 and increased cCD5 expression, respectively (Fig. 1D and E; unpaired t-test, *p* = 0.036 and* p* = 0.007, respectively).

### Identification of CEBP-β binding sites upstream of E1 A and E1B promoters of the *cd5* gene

**Fig. 2:**
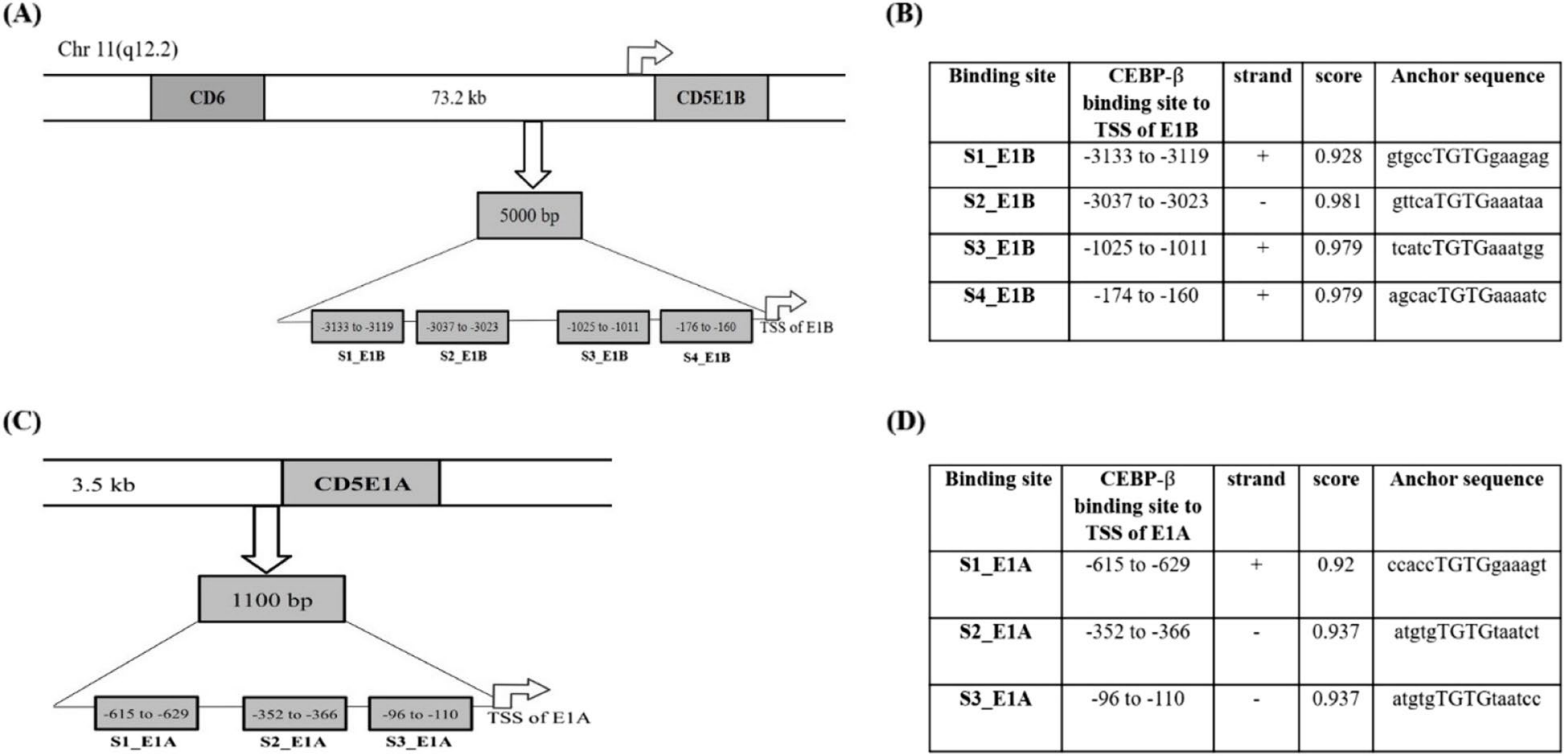
Presence of CEBP-β binding sites in the upstream sequences of E1 A and E1B variants of the ***cd5*** gene: (A) The schematic diagram shows the location of four CEBP-β binding sites present at the E1B upstream within a 5000 bp sequence from the transcription start site (TSS). **B** The table shows the exact locations of CEBP-β binding sites, the anchor sequence of each site, and their scores. These four sites were located at −3333 to −3119 (site-1, S1_E1B), −3037 to −3023 (site-2, S2_E1B), −1025 to −1011 (site-3, S3_E1B), and −174 to −160 (site-4, S4_E1B) from the farthest to the nearest site relative to the TSS of E1B variant (the farthest is numbered as S1). **C** This shows the location of three CEBP-β binding sites at the E1 A upstream within 1100 bp of the respective TSS. **B** The table shows the positions of three CEBP-β binding sites, their anchor sequences, and respective scores. These sites are denoted as S1_E1 A (−615 to −629), S2_E1 A (−352 to −366), and S3_E1 A (−96 to −110). S1_E1 A is farthest from TSS, and S3_E1 A is nearest to TSS

TFBSs were predicted within the 1100 bp upstream of the E1 A promoter and 5000 bp upstream of the non-conventional E1B promoter of the *cd5* gene using the Genomatix MatInspector Tool. Several TFBSs were identified in the upstream sequences of the E1 A and E1B variants. We narrowed it down to TFs modulated or regulated by IL-10, which are prevalent in older individuals. One of the crucial TFs we selected for further analysis was CEBP-β, and four CEBP-β binding sites were identified for the E1B promoter (Fig. 2A and B). These four sites were located at −3333 to −3119 (site-1, S1_E1B), −3037 to −3023 (site-2, S2_E1B), −1025 to −1011 (site-3, S3_E1B), and −174 to −160 (site-4, S4_E1B) from the farthest to the nearest site relative to the TSS of the E1B variant (the farthest was numbered as S1). Similarly, three CEBP-β binding sites were found in the E1 A upstream sequence (Fig. 2C and D). These sites were located at −615 to −629 (site-1, S1_E1 A), −352 to −366 (site-2, S2_E1 A), and −96 to −110 (site-3, S3_E1 A). Site-1 was distant from the TSS, and site-3 was nearest to the TSS. The identified sites had a similarity score above 0.9, indicating a high possibility of binding TFs to the site. This finding suggests that the binding of CEBP-β upstream of both TSS may regulate the expression of E1 A and E1B mRNA in older individuals. Moreover, IL-10, which is high in older cases, is known to regulate the expression of CEBP-β and its isoforms.

### IL-10 treatment upregulates CEBP-β/LIP expression at the protein level in healthy young individuals

**Fig. 3:**
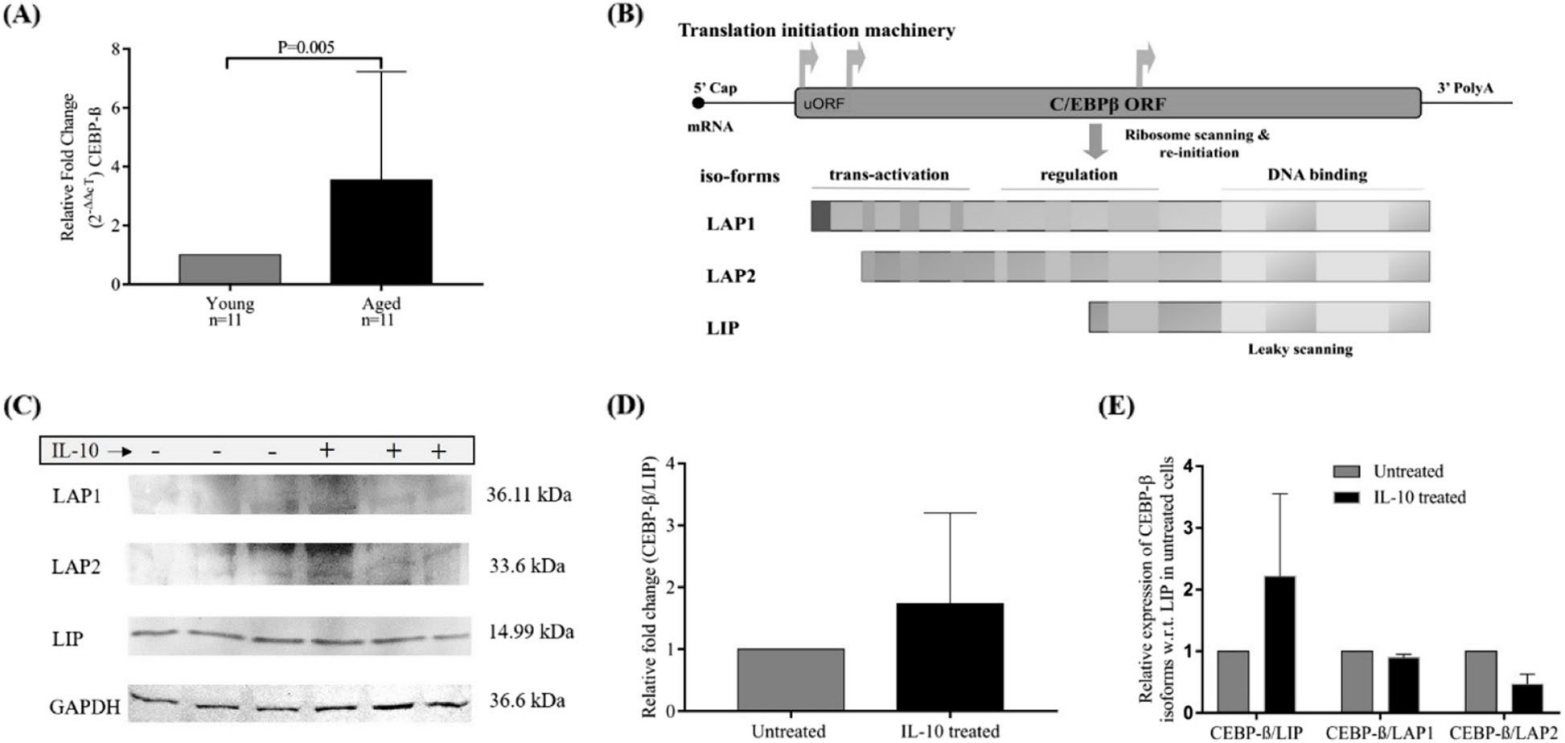
IL-10 treatment increases the level of CEBP-β/LIP isoform: (**A**) The bar diagram shows relative fold change in CEBP-β mRNA expression in peripheral blood mononuclear cells (PBMCs) of healthy young (*n* = 11) and older (*n* = 11) individuals (unpaired t-test, *p* = 0.005). **B** The schematic representation shows how a single mRNA gives rise to three isoforms (LAP1, LAP2, and LIP) owing to the presence of three different translation initiation sites, thus three open reading frames (ORF). **C** This western blot image shows the expression of three CEBP-β isoforms in untreated and IL-10-treated PBMCs from young, healthy individuals. GAPDH expression was used as a reference gene to calculate their relative fold change in expression. **D** The bar diagram shows the relative fold change in band densities of CEBP-β/LIP isoform in untreated and IL-10-treated PBMCs from young, healthy individuals (*n* = 2). **E** Relative expression (i.e., relative band intensity) of CEBP-β isoforms is shown with respect to the level of CEBP-β/LIP isoform in untreated PBMCs. Expression of U6 is used as a reference gene for calculating relative fold change in expression (2^−ΔΔcT^). Significance is shown as a *p*-value using the unpaired t-test, and values less than 0.050 are considered significant (representative of 95% CI). Control groups (young and untreated) were normalized to 1

CEBP-β is a member of the CCAAT enhancer binding protein family [36–38], and its gene contains a single exon and ultimately encodes a single mRNA. However, three isoforms, LAP1, LAP2, and LIP, were produced because of the presence of three different in-frame start codons and three independent open reading frames [39] (Fig. 3B). Older individuals showed increased expression of CEBP-β mRNA (Fig. 3A, p = 0.005). IL-10 treatment to PBMCs from healthy young individuals with a 30 ng/mL dose for 24 h showed increased expression of CEBP-β/LIP isoform using the western blotting technique (14.99 kDa; Fig. 3C). Relative fold change in expression isoform showed increased expression of CEBP-β/LIP in IL-10-treated PBMCs compared with that in untreated ones (Fig. 3D). Other isoforms [CEBP-β/LAP1 (36.11 kDA) and CEBP-β/LAP2 (33.6 kDa)] were also expressed but were quantitatively less than the inhibitory isoform CEBP-β/LIP (Fig. 3E).

### CEBP-β binds to a selected TFBS of E1 A upstream of the *cd5* gene in aged cases

**Fig. 4:**
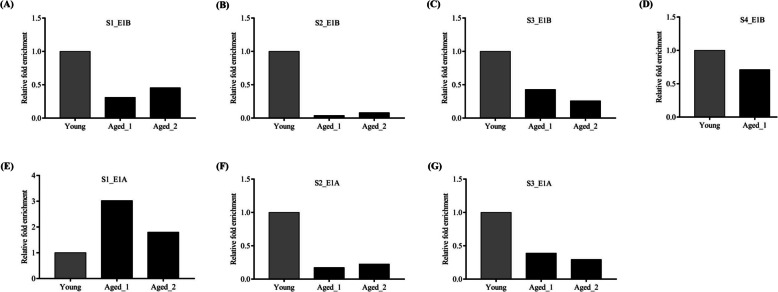
ChIP-qPCR suggesting relative fold enrichment in CEBP-β binding onto different sites in the E1 A and E1B upstream of the ***cd5*** gene:** A**-**D** The bar diagram shows the relative fold enrichments for four CEBP-β binding sites identified to E1B upstream in peripheral blood mononuclear cells (PBMCs) of young healthy (*n* = 1) and older individuals (*n* = 2). These four sites are denoted as S1_E1B (site-1, −3333 to −3119 bp), S2_E1B (site-2, −3037 to −3023), S3_E1B (site-3, −1025 to −1011), and S4_E1B (site-4, −174 to −160) from the farthest to the nearest site relative to the transcription start site (TSS) of the E1B variant. **E**–**G** Similarly, relative fold enrichments for three CEBP-β binding sites identified to E1 A upstream are shown in PBMCs of young healthy (*n* = 1) and older individuals (*n* = 2). These sites were located at S1_E1 A (site-1, −615 to −629), S2_E1 A (site-2, −352 to −366), and S3_E1 A (site-3, −96 to −110). S1_E1 A is distant from the TSS, and S3_E1 A is nearest to the TSS. Relative fold enrichment is calculated using IgG isotype as a control and depicted as a power of double delta cT (2.^−ΔΔcT^). The control group (young) was normalized to 1

When we investigated the binding of CEBP-β to all identified TFBSs upstream of the isoforms through ChIP assay, the finding showed a decrease in the enrichment of TFBS sequence of all four identified sites in the promoter of the E1B variant, suggesting lesser binding of CEBP-β to all four TFBS in freshly isolated PBMCs of older individuals than that of young individuals (Fig. 4A, S1_E1B; 4B, S2_E1B; 4 C, S3_E1B; and 4D, S4_E1B). As previously mentioned, the E1B variant was increased in older individuals, and IL-10 treatment increased the E1B mRNA variant along with the inhibitory isoform of CEBP-β/LIP in young individuals (Fig. 1B and E, respectively). Similarly, when all three identified TFBSs in E1 A upstream sequence were immunoprecipitated using an anti-CEBP-β antibody and were amplified through specific primers (i.e., ChIP assay), we observed an enrichment at S1_E1 A, i.e., better binding of CEBP-β in freshly isolated PBMCs of older individuals than that of young individuals (Fig. 4E, S1_E1 A; 4 F, S2_E1 A; and 4G, S3_E1 A). Notably, E1 A expression was reduced in older individuals and IL-10-treated cells from young individuals (Fig. 1A and D, respectively). The IgG isotype was used as a control during immunoprecipitation, and relative fold enrichment was calculated accordingly. To summarize, the binding of CEBP-β to all four sites on E1B upstream was increased in young individuals but was less in older individuals. However, at the first site in the E1 A upstream, an increased binding of CEBP-β was observed in older individuals.

### IL-10 reduces CEBP-β binding to all identified TFBSs on E1B upstream and increases CEBP-β binding to distal TFBS in E1 A upstream

**Fig. 5:**
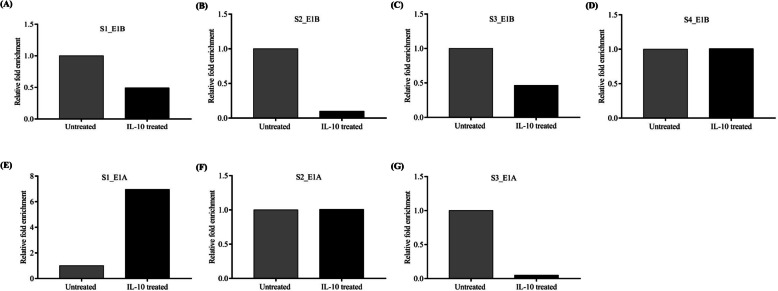
IL-10 treatment increases relative fold enrichment at S1_E1 A of the ***cd5*** gene in ChIP-qPCR assay: Relative fold enrichment for different CEBP-β binding sites in the upstream of E1B [(**A**) S1_E1B, −3333 to −3119 bp; (**B**) S2_E1B, −3037 to −3023; (**C**) S3_E1B, −1025 to −1011; and (**D**) S4_E1B, −174 to −160] and E1 A [(**E**) S1_E1 A, −615 to −629; (**F**) S2_E1 A, −352 to −366; and (**G**) S3_E1 A, −96 to −110] variants in IL-10-treated peripheral blood mononuclear cells (PBMCs) of healthy young individuals (*n* = 1, two separate repeats). S1 is distant and S3/4 is nearest to the respective transcription initiation site. Relative fold enrichment is calculated using IgG isotype as a control and depicted as a power of double delta cT (2.^−ΔΔcT^)

To investigate the role of IL-10 on CEBP-β binding to the upstream of non-conventional E1B and conventional E1 A isoforms, we performed a ChIP assay using an anti-CEBP-β antibody on PBMCs of healthy young individuals upon IL-10 treatment (30 ng/mL, 24 h). Real-time qPCR of deproteinized immune precipitate for all the TFBS sites (four for E1B and three for E1 A) revealed reduced CEBP-β binding onto the E1B upstream sequence in IL-10-treated cells (Fig. 5A, S1_E1B; 5B, S2_E1B; 5 C, S3_E1B; and 5D, S4_E1B). Similarly, ChIP assay for identified TFBSs onto E1 A upstream promoter sequence revealed increased CEBP-β binding to S1_E1 A (farthest to E1 A TSS) in IL-10-treated PBMCs of healthy young individuals (Fig. 5E, S1_E1 A). On the two sites, i.e., S2_E1 A and S3_E1 A, the binding of CEBP-β did not change or was reduced in IL-10-treated PBMCs of young individuals (Fig. 5F, S2_E1 A; and 5G, S3_E1 A). These findings are consistent with those observed in freshly isolated PBMCs from older individuals (Fig. 4E).

### shRNA silencing of CEBP-β reduces the expression of CD5 E1B

**Fig. 6:**
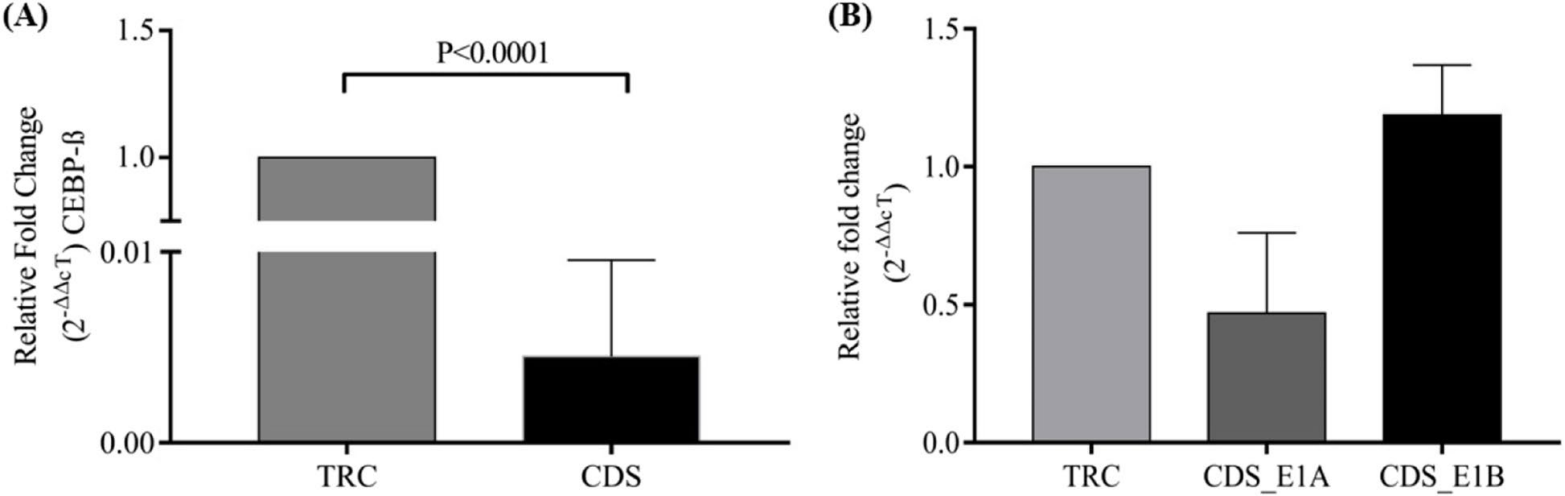
shRNA silencing experiment shows CEBP-β-mediated regulation of E1 A and E1B variant of the ***cd5*** gene: (**A**) Bar diagram shows CEBP-β mRNA levels upon lentiviral transfection for its shRNA silencing in peripheral blood mononuclear cells (PBMCs) of young individuals (*n* = 1, two separate repeats). shRNA is directed against the CDS region of CEBP-β. **B** The levels of E1 A and E1B, i.e., mRNA variants of the *cd5* gene, are shown in TRC control and CDS_shRNA PBMCs of young individuals. Expression of U6 is used as a reference gene for calculating relative fold change in expression (2^−ΔΔcT^). Significance is shown as *p*-value using the unpaired t-test, and values less than 0.050 are considered significant

We demonstrated a CEBP-β binding to the E1B promoter in PBMCs of young individuals, which was reduced in older individuals. We were inquisitive regarding whether CEBP-β binding to the E1B promoter reduced its expression in young individuals. Conversely, its binding to the E1 A upstream increases its expression in healthy young individuals. Our shRNA silencing of CEBP-β in PBMCs of young individuals using a lentiviral vector showed decreased E1 A mRNA and increased E1B mRNA expression compared with that of the TRC control (Fig. 6B). Our lentiviral vector targeted the CDS region of CEBP-β, and a significant reduction was observed after silencing (*p* = 0.0001, Fig. 6A).

## Discussion

A varying immune response, primarily due to defective T-cell function in older age, could result from unregulated cytokine production [40], reduced naïve T cells due to thymic involution [41], and, most importantly, defects in TCR signaling [42]. TCR activity depends on its interaction with its costimulatory molecules, which are adjusted by T cells themselves by changing the level of these costimulatory molecules. Modulation of the CD5 receptor, which acts as a negative regulator on the cell surface, is also responsible for the unregulated T-cell function. First, it makes them over-responsive, and second, it makes them susceptible to activation-induced cell death [43]. We have previously reported that the exon switch from conventional (i.e., E1 A exon) to non-conventional (i.e., E1B exon) is responsible for reduced sCD5 expression and increased cCD5 accumulation [22]. The absence of sCD5 is also linked to leukemic transformation, and the exon switch (E1 AE1B) in the *cd5* gene has been demonstrated as a feature of leukemic T cells. Although we were unable to directly correlate E1B upregulation in leukemia with aging, previous studies have shown that aging and leukemia share key features of immune dysregulation, including altered T-cell function and changes in CD5 expression [44]. These similarities suggest that the mechanisms underlying E1B upregulation during aging extend to leukemia, further highlighting the broader implications of our findings for immune aging and cancer.

Reduced sCD5 expression has been reported in aged T cells; however, the underlying mechanism remains unknown. In the present study, we are the first to show that an exon switch (E1 A E1B) is responsible for reduced sCD5 expression in aged T cells (Fig. 1A and 1B). This may be crucial for varying or dysregulated TCR responses in aged T cells. This may explain the chronic low-grade pro-inflammatory state in many older individuals. CD5 blocking unleashes the capability of T cells to produce inflammatory cytokines in response to polyclonal/CD3 stimulation, suggesting an overresponsive CD5^low/neg^ T-cell phenotype. This nonspecific, persistent, chronic, and mild inflammation in older individuals is a potential risk factor for the onset and progression of many degenerative diseases. In older individuals, this state is referred to as “inflammaging.” Inflammaging is characterized by the dominance of pro-inflammatory cytokines and a significant presence of anti-inflammatory cytokines, such as IL-10 [45], which is paradoxical. However, the overall manifestation of the response remained slightly inflammatory at a given site in aged individuals. Aged T cells produce high levels of IL-10 owing to their high avidity toward costimulatory molecules or MHC-induced chronic TCR engagement [46]. In this study, we confirmed that older individuals had elevated IL-10 levels (Fig. 1C), which we hypothesized could contribute to the E1 A → E1B switch. In support of this, when young PBMCs were treated with IL-10, a shift toward increased E1B expression and reduced E1 A expression was observed (Fig. 1D and 1E). Our further investigations into the TFs involved in this exon switch revealed that CEBP/β, an immune-regulatory TF involved in age-related physiological changes, plays a central role in modulating the observed exon switch.

Moreover, IL-10-mediated regulation of CEBP/β and its isoforms has already been reported [47]. Further, our finding suggests that IL-10-driven increase in CEBP/β, particularly its inhibitory isoform (CEBP/β-LIP), contributes to the suppression of E1 A expression and the promotion of E1B expression.

IL-10 promotes the increase of the LIP isoform of CEBP/β, which binds to the first site upstream of the E1 A TSS, suppressing E1 A expression and enhancing E1B expression. ChIP assays revealed decreased CEBP/β binding to the E1B upstream in aged and IL-10-treated young PBMCs, confirming the involvement of the CEBP/β-LIP isoform in this regulation. Silencing CEBP/β with shRNA in young PBMCs led to increased E1B expression, further supporting the role of IL-10 in driving the E1 A → E1B exon switch in aged T cells.

IL-10 is recognized for its anti-inflammatory properties and plays a key role in regulating immune responses by inhibiting pro-inflammatory cytokine production. However, in the context of aging, our findings suggest that IL-10 also contributes to immunopathology by promoting the E1 A → E1B exon switch in CD5, a shift that leads to reduced sCD5 expression on T cells. This reduction in CD5 expression may impair the immune response, thereby facilitating immunosenescence. These dual functions of IL-10 highlight the complexity and the need to consider its immunosuppressive and potential immunopathological effects on the aging immune system. The clinical significance of these findings lies in their potential implications in immunosenescence and immune-related diseases. This dysregulated immune phenotype may explain the persistent low-grade inflammation observed during aging, which is a risk factor for the onset and progression of degenerative diseases. Moreover, these findings provide new insights into the mechanisms of immune dysfunction in hematologic malignancies such as T-ALL and chronic lymphocytic leukemia, where immune dysregulation, including altered CD5 expression, is a common feature.

The dual role of IL-10 in immune regulation (anti-inflammatory and potentially pro-inflammatory) in aging highlights the complexity of cytokine signaling in the context of aging. Understanding this duality is crucial for developing immunotherapeutic strategies targeting IL-10 or its signaling pathways for chronic inflammatory diseases and cancer.

Overall, our study reveals the E1 A → E1B switch as a key mechanism regulating CD5 expression in aging and underscores the broader implications of this switch in immunosenescence and immune dysfunction in aging and cancer. This highlights the importance of IL-10 as a potential therapeutic target in age-related immune pathologies and cancer immunotherapy, particularly for inflammaging. However, our study has certain limitations, including the sample size, the absence of isoform-specific protein-level validation for CEBP/β knockdown, and the semi-quantitative nature of the ChIP assay. Additionally, owing to limited sample availability, some experiments, such as ChIP-PCR and qPCR validation, were conducted with minimal technical replicates. These factors should be considered when interpreting our results and drawing broader conclusions.

## Supplementary Information

Supplementary Material 1.

## Data Availability

No datasets were generated or analysed during the current study.
