# Supplementary material for: Interleukin-10 augments human endogenous retroviral E1B variant of cd5 in aged T cells
Source: Blood Res. 2025 Aug 11;60(1):43. doi: 10.1007/s44313-025-00080-8 (PMC12339811; doi:10.1007/s44313-025-00080-8)
Supplement: Supplementary file 1 — Supplementary Material 1. [file 44313_2025_80_MOESM1_ESM.docx]

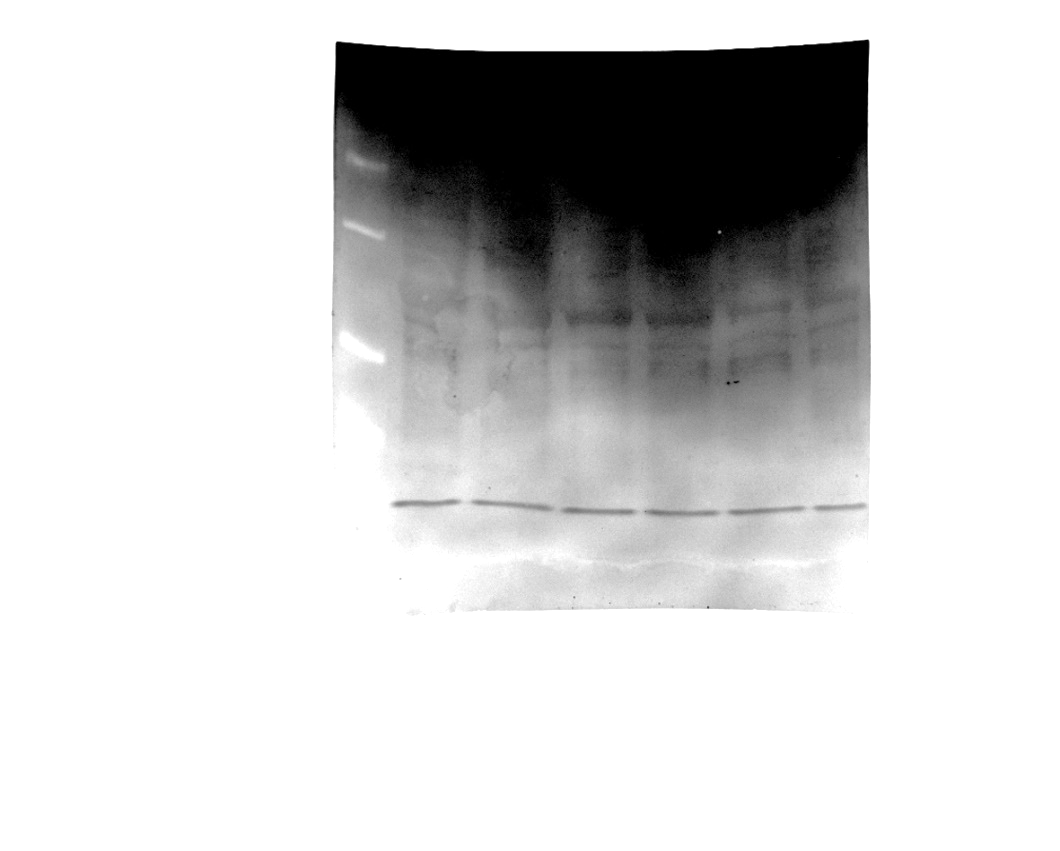

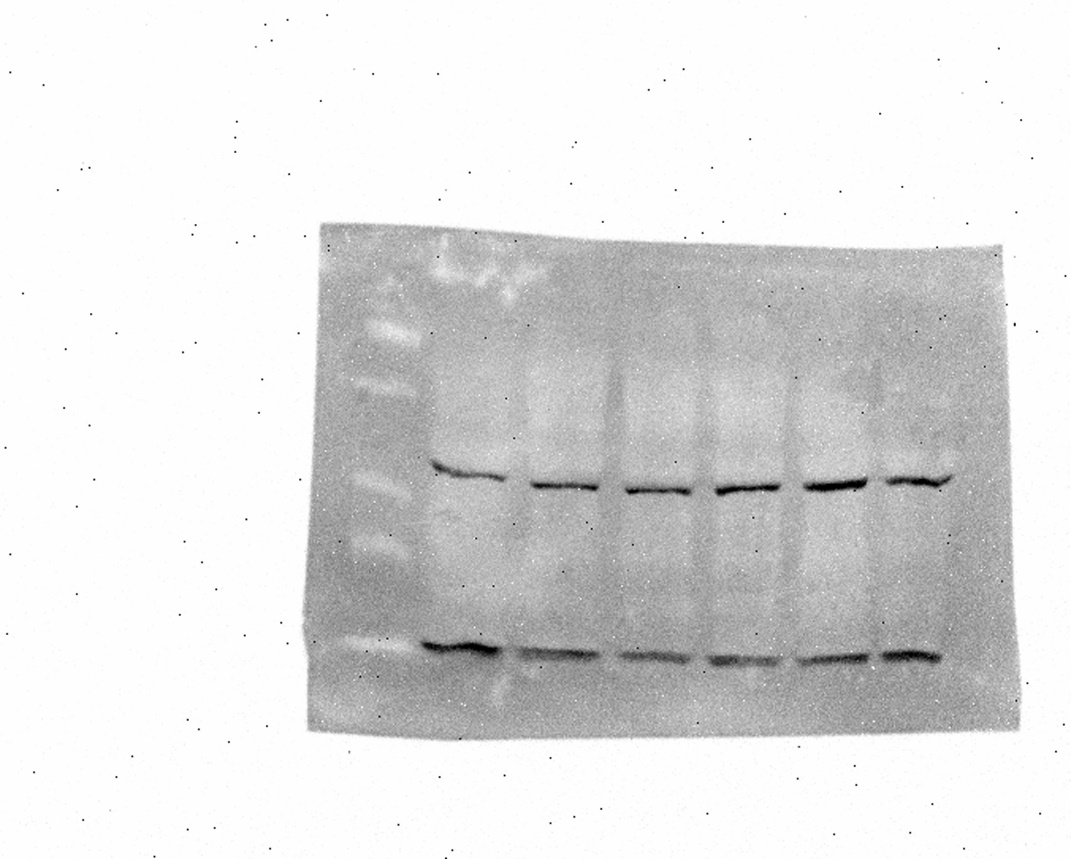


55KDda

35KDda

35KDda

55KDda

Ladder

Ladder

LAP1 (CEBP-β)

LAP2 (CEBP-β)

LAP1 (CEBP-β)

Supplementary figure: Western blot images for checking expression of CEBP-β in human blood PBMC samples

GAPDH
